# Intensity-modulated radiotherapy combined with systemic atezolizumab and bevacizumab in treatment of hepatocellular carcinoma with extrahepatic portal vein tumor thrombus: A preliminary multicenter single-arm prospective study

**DOI:** 10.3389/fimmu.2023.1107542

**Published:** 2023-02-16

**Authors:** Kang Wang, Yan-Jun Xiang, Hong-Ming Yu, Yu-Qiang Cheng, Zong-Han Liu, Jing-Ya Zhong, Shuang Feng, Qian-Zhi Ni, Hong-Fei Zhu, Wei-Wei Pan, Jing-Jing Li, Chao Liang, Hong-Kun Zhou, Yan Meng, Wan Yee Lau, Shu-Qun Cheng

**Affiliations:** ^1^ Department of Hepatic Surgery VI, Eastern Hepatobiliary Surgery Hospital, Naval Medical University, Shanghai, China; ^2^ Department of Cell Biology, College of Medicine, Jiaxing University, Jiaxing, China; ^3^ Department of Radiotherapy, Eastern Hepatobiliary Surgery Hospital, Naval Medical University Shanghai, Shanghai, China; ^4^ CAS Key Laboratory of Nutrition, Metabolism and Food Safety, Shanghai Institute of Nutrition and Health, University of Chinese Academy of Sciences, Chinese Academy of Sciences, Shanghai, China; ^5^ G60 STI Valley Industry & Innovation Institute, Jiaxing University, Jiaxing, China; ^6^ Yueyang Hospital of Integrated Traditional Chinese and Western Medicine, Shanghai University of Traditional Chinese Medicine, Shanghai, China; ^7^ The First Hospital of Jiaxing Affiliated Hospital of Jiaxing University, Jiaxing University, Jiaxing, China; ^8^ Faculty of Medicine, The Chinese University of Hong Kong, Hong Kong, Hong Kong SAR, China

**Keywords:** PD-L1 inhibitor, immunotherapy, intensity-modulated radiotherapy, combination therapy, macrovascular invasion

## Abstract

**Background and aims:**

The efficacy and safety of systemic atezolizumab and bevacizumab (atezo/bev) in treatment of patients with unresectable hepatocellular carcinoma (HCC) have been demonstrated. However, the efficacy of this treatment in patients with HCC and extrahepatic portal vein tumor thrombus (ePVTT) is not satisfactory. This study aimed to study the efficacy and safety of combining intensity-modulated radiotherapy (IMRT) with systemic atezo/bev in treatment of these patients.

**Methods:**

This multicenter prospective study included patients with ePVTT treated with IMRT combined with atezo/bev from March to September 2021 in three centers in China. The outcomes of this study included objective response rate (ORR), overall survival (OS), progression-free survival (PFS), time to progression (TTP), and association between response and tumor mutational burden (TMB). Treatment-related adverse events (TRAEs) were analyzed to assess safety.

**Results:**

Of 30 patients in this study, the median follow-up was 7.4 months. Based on the Response Evaluation Criteria in Solid Tumors (RECIST) version 1.1, the ORR was 76.6%, the median OS for the entire cohort was 9.8 months, the median PFS was 8.0 months, and the median TTP was not reached. This study failed to establish a significant correlation between TMB with any of the following outcomes, including ORR, OS, PFS or TTP. The most common TRAEs at all levels were neutropenia (46.7%), and the most common grade 3/4 TRAE was hypertension (16.7%). There was no treatment-related deaths.

**Conclusions:**

IMRT combined with atezo/bev showed encouraging treatment efficacy with an acceptable safety profile, making this treatment to be a promising option for HCC patients with ePVTT. Further studies are required to support the findings of this preliminary study.

**Clinical trial registration:**

http://www.chictr.org.cn, Identifier ChiCTR2200061793.

## Introduction

Hepatocellular carcinoma (HCC), a primary liver cancer, has been a hot topic in cancer research because of its high morbidity and mortality (1). Approximately 10 to 40% of HCC patients present with portal vein tumor thrombus (PVTT) at the time of diagnosis. The prognosis of these patients is poor, and the median overall survival (OS) ranges from 2.7 to 4.0 months (2). Treatment of HCC patients with PVTT remains a great challenge.

Oral administrations of sorafenib or lenvatinib are the standard first-line treatment for patients with advanced HCC, but prognosis is not satisfactory, especially in patients with PVTT (3–6). A recent clinical trial (IMbrave150) showed that combined systemic atezolizumab with bevacizumab (atezo/bev) gave better treatment survival outcomes for patients with unresectable HCC when compared with sorafenib (7–9). The astounding efficacy of atezo/bev has made it a possibility to be used as a first-line therapy for advanced HCC. Even for HCC patients with PVTT, atezo/bev therapy has been shown to improve median OS of these patients (10, 11).

Systemic therapy combined with locoregional therapy has been shown to improve prognosis of HCC patients with PVTT (12, 13). Of the locoregional therapies, intensity-modulated radiotherapy (IMRT) has shown great promise in local control of PVTT (14, 15). A previous randomized controlled trial from our group demonstrated that preoperative neoadjuvant three-dimensional conformal radiotherapy significantly improved outcomes in patients with PVTT (16). Interestingly, PVTT is more sensitive to radiotherapy as compared to the primary tumor (17). A toxicological study of atezo/bev combo in the treatment of patients with unresectable HCC using atezo/bev combined with radiotherapy to the tumor showed the combined treatment was well tolerated with a high safety profile (18). Therefore, combining IMRT can be a more effective treatment for HCC patients with PVTT.

In this prospective study, the efficacy and safety of systemic atezo/bev combined with IMRT in HCC patients with extrahepatic PVTT (ePVTT), and novel biomarkers in predicting response to treatment by genomic profiling, were studied.

## Patients and methods

### Patients selection

Patients with HCC and ePVTT treated with atezo/bev combined with IMRT from March to September 2021 at Eastern Hepatobiliary Hospital, Yueyang Hospital of Integrated Traditional Chinese and Western Medicine, and the First Hospital of Jiaxing Affiliated Hospital of Jiaxing University were prospectively studied. The inclusion criteria were patients with (1) HCC with ePVTT as defined in the subsequent section of this article (2); age over 18 years (3); unresectable HCC as assessed by experienced liver surgeons (4); good general status and liver function of Pugh-Child A or B. The exclusion criteria were patients with (1) contraindications to atezo/bev or IMRT (2); prior HCC treatment (3); history of other cancers (4); pregnancy. Patients with extrahepatic spread of HCC was not a contraindication of this study.

This study was carried out in compliance with the ethical standards of Declaration of Helsinki. It was approved by the Institutional Ethics Committee of each center. Informed consent was obtained from all patients prior to treatment and for their data to be used in clinical research. This study is registered with the Chinese Clinical Trial Registry, ChiCTR2200061793.

### Diagnosis and treatment

In this study, HCC was diagnosed based on alpha fetoprotein levels, dynamic computed tomography, dynamic magnetic resonance imaging, or pathology findings (5, 19). PVTT was diagnosed based on the European Association for the Study of the Liver (EASL) Guidelines and the Chinese Expert Consensus on Multidisciplinary Diagnosis and Treatment of PVTT (19, 20). PVTT was staged according to the Cheng’s classification and the portal vein invasion (Vp) classification (21–23). Extrahepatic PVTT (ePVTT) was defined as PVTT which had extended extrahepatically to involve either the right or left portal vein (Rt/Lt PV), the main portal vein (MPV) and the superior mesenteric vein (SMV). High-risk status was defined as PVTT invasion of MPV and/or SMV (Vp4), and/or tumor occupancy of ≥ 50% of liver (10, 11).

After a multidisciplinary consultation to include patients who were considered to be appropriate to receive atezo/bev combined with IMRT, these patients were fully informed about the potential efficacy of the combined treatment, treatment-related adverse events (TRAEs) and costs of treatment.

IMRT was first given to these patients as previously reported (16). Each radiotherapy cycle was 28 days, with a planned total dose of 52-56 Gy on the planned target area of ePVTT at 200 cGy/dose. Systemic atezo/bev was initiated 3 ± 1 days after completion of radiotherapy, and each cycle of systemic therapy was 21 days. A fixed dose of 1200 mg of atezolizumab was administered intravenously on the first day of each systemic therapy cycle (60 min for the first session, 30 min for subsequent sessions if tolerated), and bevacizumab 15 mg/kg was administered intravenously at least 5 min apart (90 min for the first session, 30 min for subsequent sessions if tolerated). Treatment continued until unacceptable toxicity or loss of clinical benefit as assessed by investigators based on imaging findings, biochemical parameters and patient’s clinical status.

### Follow-up and outcomes

All patients were followed-up in the outpatient clinic once every 6 weeks. At each follow-up visit, there were routine history taking, physical examination, laboratory blood tests, and abdominal ultrasound or enhanced CT/MRI. Assessment of tumor progression was based on the Response Evaluation Criteria in Solid Tumors (RECIST) version 1.1 and the modified RECIST (mRECIST).

The primary outcome of this study was the objective response rate (ORR) which was defined as the proportion of patients in complete response (CR) and partial response (PR). The secondary outcomes included OS, progression-free survival (PFS), and time to progression (TTP). TRAEs were assessed based on the criteria of Common Terminology Criteria for Adverse Events, version 5.0. For multiple instances of the same toxicity for each patient, the highest grade in a given category was adopted.

### Definition of outcomes

Tumor responses were evaluated as previously reported (16). Briefly stated, patients were diagnosed to have progressive disease (PD) when either the primary tumor or ePVTT was evaluated as PD; stable disease (SD) when both lesions were evaluated as SD; PR when either one of these two lesions was evaluated as PR; and complete response (CR) when both lesions were evaluated as CR. PVTT response was defined according to the upstaging or downstaging in Cheng’s PVTT classification. Patients were treated with optimal treatments based on their general condition, liver function and extent of HCC after PD.

OS was defined as the time from the first study treatment to the date of death from any cause, or the date of the last follow-up. PFS was defined as the time from the first study treatment to tumor progression, death from any cause, or the last follow-up. TTP was defined as the time from the first study treatment to the appearance of any objective evidence of tumor progression. Tumor progression was assessed based on RECIST version 1.1.

### Biomarker analysis

Optional baseline biopsy specimens were obtained from patients for exploratory biomarker evaluation. Specifically, genomic DNA was extracted from tumor tissues, followed by next-generation sequencing and analysis of tumor mutation burden (TMB). Differences in gene mutations between the response group (patients with CR or PR) and the non-response group (patients with SD or PD) were compared.

### Statistical analysis

All clinical data were analyzed using IBM SPSS Statistics 23 and R 4.0.2 software (http://www.r-project.org/). In baseline variables considered as binary variables, the cutoff values were defined based on uniform consensus. The optimal cutoff value of TMB was calculated using the “pROC” package of R based on the maximum Youden index to distinguish high from low TMB. OS, PFS and TTP were estimated using the Kaplan-Meier method and compared with the log-rank test. The Fisher exact test was used to compare variables, and a P < 0.05 was defined as significantly different.

## Results

### Patients

**Figure 1:**
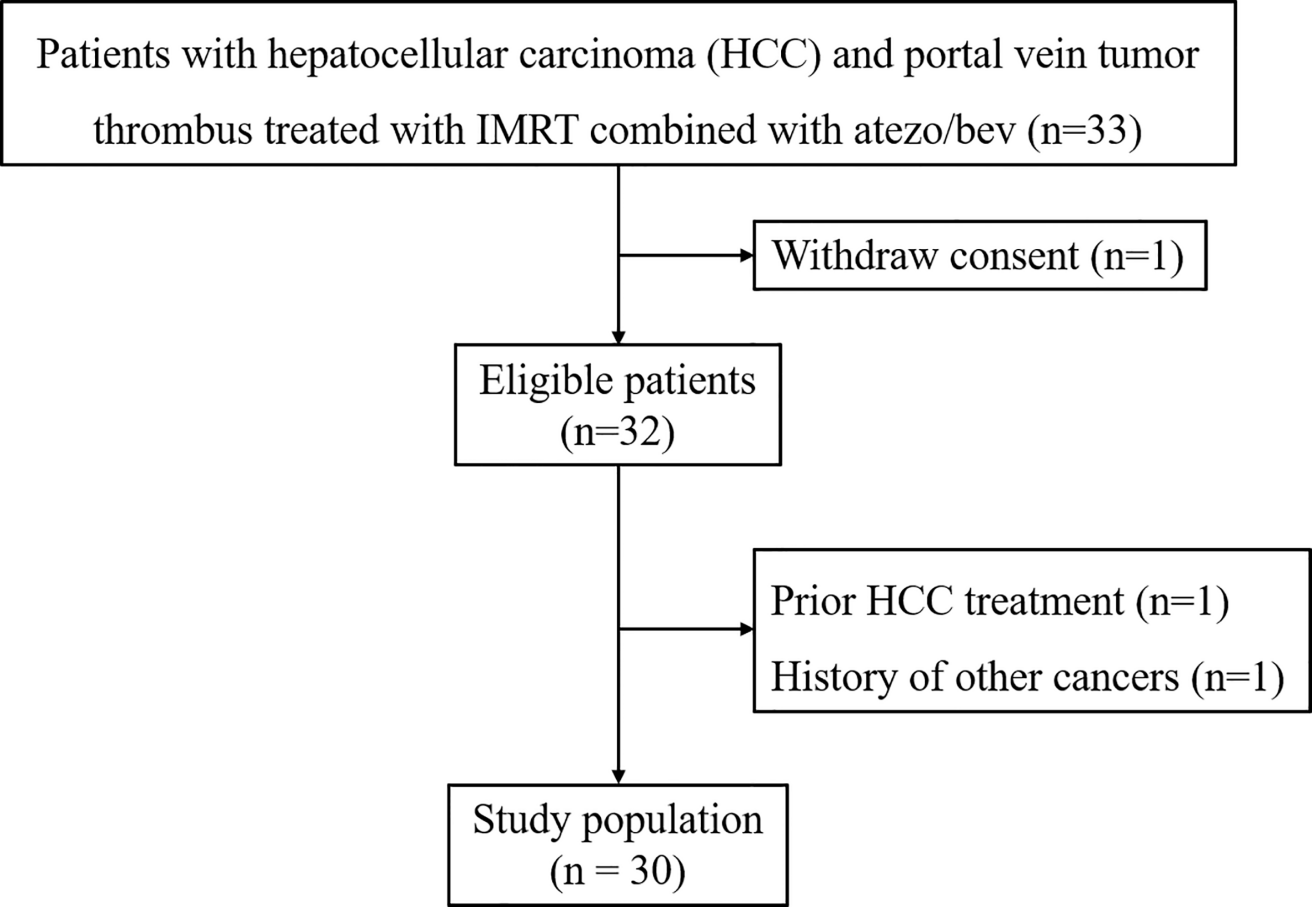
Patient Flow Diagram. Atezo/bev, atezolizumab and bevacizumab; IMRT, intensity-modulated radiotherapy.

Thirty patients were included into this study (**Figure 1**). The clinicopathological characteristics of these patients are summarized in **Table 1**. The median age of a total of 30 patients was 56.5 years (range, 37-38 years), of whom 25 patients (83.3%) were male, 26 patients (86.7%) had co-infection with HBV, and 25 patients (83.3%) with cirrhosis. Eighteen patients (60.0%) had ePVTT extending to the MPV or SMV while 12 patients (40%) had ePVTT involving just the Rt/Lt PV. Concomitant extrahepatic metastases were present in 16 patients (53.3%), and combined hepatic vein carcinoma thrombus was present in nine patients (30.0%). Twenty-nine patients were at high-risk status, 11 of them with tumor ≥50% of the liver, six with PVTT extending to MPV or SMV, and 12 with both of these risk factors. On March 16, 2022 when this study was censored, and no patient withdrawals. The median follow-up for the entire cohort was 7.4 months, and the median cycle of atezo/bev was eight.

**Table 1:** Baseline characteristics of study patients.

Characteristics	All (n=30)
Age, years, median (range)	56.5 (37-83)
Gender, male	25 (83.3)
ECOG PS, 0/1	30 (100.0)
Child-Pugh score, A	23 (76.7)
ALBI grade, 2	21 (70.0%)
Etiology	
HBV	26 (86.7)
HCV	1 (3.3)
Nonviral	3 (10.0)
Liver cirrhosis, yes	25 (83.3)
Varices, yes	22 (73.3)
AFP, ng/mL, ≥ 400	18 (60.0)
DCP, mAU/mL, ≥ 2050	19 (63.3)
Tumor number, Multiple	24 (80.0)
Tumor of the liver, ≥50%	23 (76.7)
Extrahepatic spread	
Lung	7 (23.3)
Lymph node	8 (26.7)
Bone	1 (3.3)
Hepatic vein tumor thrombus, With	9 (30.0)
With	9 (30.0)
PVTT location, mPV/SMV (VP4)	18 (60.0)
High-risk status^*^, yes	29 (96.7)

ECOG PS, Eastern Cooperative Oncology Group performance status; ALBI grade, albumin-bilirubin grade; Rt/Lt PV, right or left portal vein; mPV, main portal vein; SMV, superior mesenteric vein. * High-risk status was defined as PVTT invasion of MPV and/or SMV (Vp4), and/or tumor occupancy of ≥ 50% of liver.

### Treatment efficacy

**Table 2** summarizes the best tumor response for all patients. Based on RECIST version 1.1, the ORR was 76.6% and the disease control rate (DCR) was 96.7%. On subgroup analysis, the ORR and DCR were 75.0% and 100% for ePVTT in Rt/Lt PV, and 77.8% and 94.4% for ePVTT extending to MPV or SMV, respectively. Based on mRECIST, the ORR and DCR for the entire cohort were 90.0% and 96.7%, respectively, and the ORR and DCR for ePVTT in Rt/Lt PV were 91.7% and 100%, respectively, and 88.9% and 94.4% for ePVTT extending to MPV or SMV, respectively.

**Table 2:** Best tumor response.

	All patients(n=30)	PVTT location		P value
		Rt/Lt PV (n=12)	mPV/SMV (n=18)	
RECIST 1.1				
CR	2	2	0	
PR	21	7	14	
SD	6	3	3	
PD	1	0	1	
ORR	76.6%	75.0%	77.8%	1.000
DCR	96.7%	100.0%	94.4%	1.000
mRECIST				
CR	4	3	1	
PR	23	8	15	
SD	2	1	1	
PD	1	0	1	
ORR	90.0%	91.7%	88.9%	1.000
DCR	96.7%	100.0%	94.4%	1.000

Rt/Lt PV, right or left portal vein; mPV, main portal vein; SMV, superior mesenteric vein; CR, complete response; PR, partial response; SD, stable disease; PD, progressive disease; ORR, objective response rate; DCR, disease control rate.

**Figure 2:**
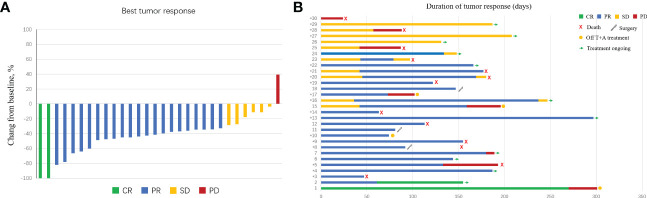
Characteristics of objective response in patients with intensity-modulated radiotherapy (IMRT) radiotherapy combined with atezolizumab plus bevacizumab. **(A)** The maximum percentage reduction from baseline in primary tumor. **(B)** Duration of response.

**Figure 3:**
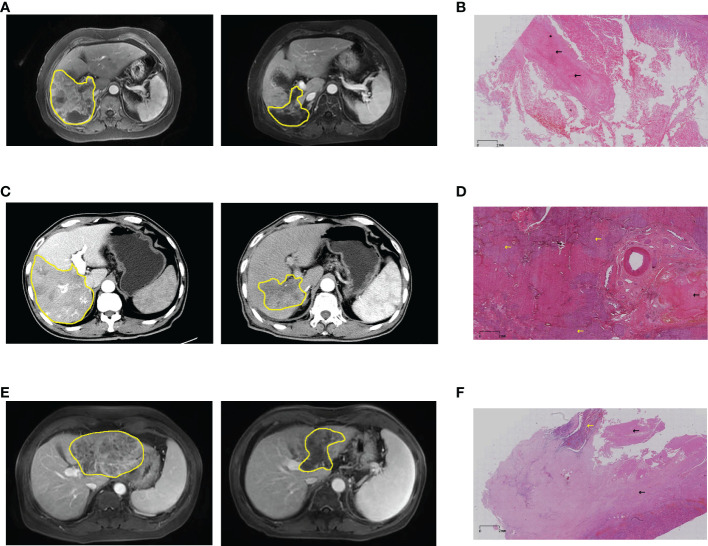
Imaging and pathological information of patients who underwent hepatectomy. **(A)** Pre- and post-treatment MRI of patient #8, and **(B)** post-operative pathology showed pathological complete response. **(C)** Pre- and post-treatment MRI of patient #11, and **(D)** postoperative pathology showed partial necrosis. **(E)** Pre- and post-treatment MRI of patient #18, and **(F)** postoperative pathology showed a large area of coagulative necrosis (80%). Yellow lines and arrows for hepatocellular carcinoma, black arrows for coagulative necrosis. MRI, magnetic resonance imaging.

Based on RECIST version 1.1, 29 patients had a reduction in size of the primary tumor when compared to baseline (**Figure 2**). Details of the response durations and outcomes are presented in **Figure 2**. Of the 30 patients in this study, 13 patients had died during follow-up (suspected causes of death are detailed in **Supplemental Table 1**). Eleven surviving patients continued to receive atezo/bev, while three patients had discontinued atezo/bev (two due to tumor progression and one due to financial burden). Three patients underwent liver resection aiming at cure after tumor-downstaging. The imagings and pathological information of the 3 operated patients are shown in **Figure 3**.

**Figure 4:**
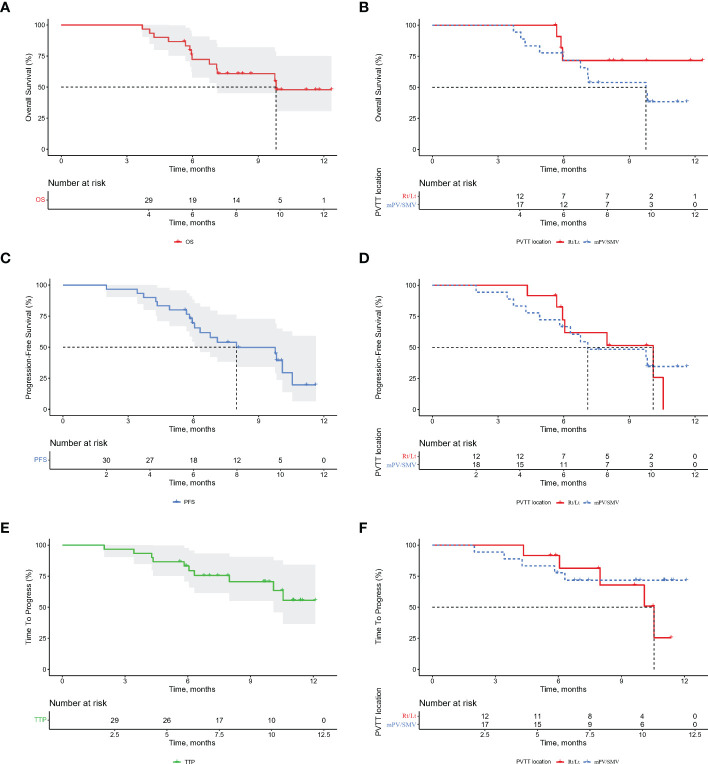
Kaplan-Meier curves for overall survival (OS), progression-free survival (PFS), and time to progression (TTP) for the entire cohort of patients **(A, C, E)** and portal vein tumor thrombus subgroups **(B, D, F)**. Rt/Lt, right or left portal vein; MPV, main portal vein; SMV, superior mesenteric vein.

The median OS was 9.8 months for the entire cohort (**Figure 4**), not reached for ePVTT in Rt/Lt PV and 9.8 months for ePVTT extending to MPV or SMV (**Figure 4**). The median PFS was 8.0 months for the entire cohort (**Figure 4**), 10.1 and 7.1 months for ePVTT in Rt/Lt PV and ePVTT extending to MPV or SMV, respectively (**Figure 4**); the median TTP was not reached for the entire cohort (**Figure 4**), 10.5 months for ePVTT in Rt/Lt PV, and was not reached for ePVTT extending to MPV or SMV (**Figure 4**).

To better compare the results with those of the IMbrave150, patients with high-risk status were analyzed. The results showed 29 high-risk patients to have a median OS of 9.8 months and a median PFS of 8.0 months (**Supplemental Figure 1**).

### Safety

TRAEs during treatment based on their frequency and severity were evaluated using the CTCAE version 5.0. As shown in **Table 3**, the most common TRAEs at all levels were neutropenia (46.7%), fatigue (40.0%) and hypertension (33.3%), and the most common grade 3/4 TRAEs were hypertension (10.0%) and aspartate aminotransferase elevation (10%). No patient developed classical radiation-induced liver disease, and there was no treatment-related death.

**Table 3:** Treatment emergent adverse events.

Adverse Events	All (n=30)		Rt/Lt PV (n=12)	mPV/SMV (n=18)
	Any grade, n (%)	Grade 3/4. n (%)	Grade 3/4. n (%)	Grade 3/4. n (%)
Any adverse event	29 (97)	8 (27)	2 (17)	6 (33)
Fatigue	12 (40)	0	0	0
Pruritus	2 (7)	0	0	0
Rash	4 (13)	0	0	0
Anorexia	6 (20)	0	0	0
Nausea	4 (13)	0	0	0
Vomiting	3 (10)	1 (3)	0	1 (6)
Diarrhoea	3 (10)	0	0	0
Hypertension	10 (33)	3 (10)	1 (8)	2 (11)
Proteinuria	1 (3)	1 (3)	1 (8)	0
Constipation	1 (3)	0	0	0
Hypothyroidism	3 (10)	0	0	0
Immune-mediated hepatitis	3 (10)	1 (3)	0	1 (6)
GI haemorrhage	2 (7)	2 (7)	1 (8)	1 (6)
GI tract perforation	/	1 (3)	0	1 (6)
Neutropenia	14 (47)	0	0	0
Anaemia	7 (23)	1 (3)	0	1 (6)
Thrombocytopenia	9 (30)	1 (3)	1 (8)	0
AST elevation	8 (26)	3 (10)	1 (8)	2 (11)
ALT elevation	6 (20)	2 (7)	0	2 (11)
Hyperbilirubinemia	7 (23)	1 (3)	0	1 (6)
Progression of Child-Pugh score ≥ 2	9 (30)		4 (33)	5 (28)

There was no significant difference in grade 3/4 adverse events between the Rt/Lt PV and mPV/SMV groups (P=0.419).

ALT, alanine aminotransferase; AST, aspartate aminotransferase; GI, gastrointestinal tract; Rt/Lt PV, right or left portal vein; mPV, main portal vein; SMV, superior mesenteric vein.

### Biomarkers

Pathological tissues were obtained from 15 of 30 HCC patients, including specimens from 12 PR patients and 3 SD patients. These optional biopsy specimens were obtained for exploratory biomarker assessment. Based on tumor response to treatment, 228 mutations/megabase were used as the threshold of TMB to distinguish between high and low TMB. The results showed that ORR was 66.7% in the high TMB group and 100% in the low TMB group, and there was no significant difference between the two groups (**Supplemental Figure 2**, P=0.229). No association was observed between TMB with OS (HR=0.815, 95%CI= 0.215-3.082, P=0.763, **Supplemental Figure 3**), with PFS (HR=0.663, 95%CI= 0.200-2.202, P=0.502, **Supplemental Figure 3**) or with TTP (HR=0.335, 95%CI= 0.059-2.142, P=0.259, **Supplemental Figure 3**). **Supplemental Figure 2** depicts the distributions of genetic variation which were associated with response to the combined treatment using atezo/bev and IMRT. The most frequently altered genes in the response group when compared with the non-response group were CARS1P1, CCDC146, GLI2, SART3 and SSU72P3 (P<0.05).

## Discussion

The efficacy and toxicity of IMRT in combination with systemic atezo/bev in 30 HCC patients with ePVTT were studied. The ORR of entire cohort was 76.6%, with a median OS and PFS of 9.8 and 8.0 months, respectively. To our knowledge, this is the first reported study of using combined IMRT with atezo/bev to treat HCC patients with ePVTT.

HCC with PVTT is generally considered to be at an advanced stage of disease. Although there are still some controversies on whether PVTT affecting secondary and tertiary branches of the main portal vein should be treated with liver resection, there is little controversy that once the extrahepatic portal venous system is affected by PVTT (ePVTT), systemic therapy is recommended by treatment guidelines as the standard of care for these patients (19, 20, 24–26). Tumor invasion into the portal venous system not only promotes intrahepatic tumor spread of the disease, but when the extrahepatic portal venous system is involved, the portal blood supply to the liver is rapidly reduced, resulting in rapid deterioration of liver function, and increase in portal pressure with its risks of complication, which can lead to further limitations of choice of treatment options (27). Any measures that can lead to a reduction in ePVTT volume can result in increasing portal blood supply to the liver and decrease in portal hypertension, thus facilitating subsequent choice of treatment for HCC patients with ePVTT. External radiation has long been used as a local-regional treatment of HCC. A recent study reported that PVTT was more sensitive to radiotherapy than the primary HCC (17, 28–30). A recent open-label randomized clinical trial showed that TACE combined with external beam radiotherapy provided better PFS, ORR, TTP, and OS when compared with sorafenib in HCC patients with PVTT (31), and external radiotherapy has been shown to be more effective than sorafenib in treatment of HCC patients with ePVTT (5). In addition, as external radiotherapy can trigger immunogenic cell death resulting in release of cytokines and damage-associated molecular patterns (DAMPs), subsequent priming and trafficking of tumor-specific T lymphocytes into the tumor microenvironment by DAMPs can enhance recruitment of antigen-presenting cells, processing of tumor-associated antigens, and cross presentation of antigenic peptides on major histocompatibility complex class I, thereby improving the efficacy of immunotherapy (32). All these provide the theoretical basis for the combination therapy of IMRT with atezo/bev in treatment of HCC patients with ePVTT.

The extent of HCC in this study was advanced, as evidenced by almost all patients (96.7%) were at high-risk status. In this study, the median OS and PFS for such patients were 9.8 and 8.0 months, respectively, which were higher than the 7.6 and 5.4 months in the IMbrave 150 (10). A better oncological prognosis was also observed in the group of patients with PVTT extending to MPV or SMV (11). Considering the small sample size of this study, no definitive conclusions can be drawn that this treatment regimen is superior to IMbrave 150. However, the data supports that this new treatment improved prognosis of HCC patients with ePVTT. This treatment has also been shown to be safe, with the most common adverse effect being hypertension, similar to the results previously reported (7, 33). As gastrointestinal bleeding and perforation have been reported to occur on using bevacizumab, 2 patients in our study developed grade 3 or higher gastrointestinal bleeding, a rate which was higher than those previously reported on using only atezo/bev (7, 33). This may be due to the fact that most of the included patients with VP4 PVTT (34). However, no patients died from treatment-related side effects which was unexpected given the relatively advanced HCC stages of our patients who had also received IMRT (35, 36). Esophagogastroduodenoscopy should be used as a routine before patients receiving treatment using combined IMRT and atezo/bev.

This study on using biomarkers failed to establish any correlations between TMB with any one of the following treatment outcomes, including ORR, OS, PFS or TTP. These findings are consistent with the results of previously reported studies on immunotherapy (37, 38). Instead, the tumor response seemed to be associated with alterations in some specific genes. Overexpression of GLI2 has been reported to cause resistance to targeted cancer therapy in cancer cells, and another study reported that knockdown of GLI1 and GLI2 restored sensitivity to vemurafenib-resistant cells (39, 40). However, the small sample size of our study limited further analysis of these reported findings, and clinical utility of these findings remains to be further studied in the future.

There are several limitations of the study. First, this is multicenter study on a small sample size of patients. Second, the short follow-up was not sufficient to assess long-term survival outcomes in patients treated with IMRT and atezo/bev. As a consequence, ORR was used as the primary outcome. Third, this study was conducted in China where hepatitis B infection is endemic. The results of this study may not be applicable to HCC patients seen in the West where etiologies of HCC are mainly due to hepatitis C infection, alcoholism and non-alcoholic steatohepatitis.

In conclusion, combined IMRT with atezo/bev resulted in encouraging treatment efficacy and acceptable safety, making the treatment to be a promising option for patients with HCC and ePVTT. Comprehensive genomic analysis of mutation profile involving multiple biomarkers helps in providing a framework for molecularly stratified treatment of advanced HCC in future studies.

## Data availability statement

The raw data supporting the conclusions of this article will be made available by the authors, without undue reservation.

## Ethics statement

The studies involving human participants were reviewed and approved by the Institutional Ethics Committees of the Eastern Hepatobiliary Hospital. The patients/participants provided their written informed consent to participate in this study. Written informed consent was obtained from the individual(s) for the publication of any potentially identifiable images or data included in this article.

## Author contributions

Conceptualization: S-QC and KW. Funding acquisition: S-QC and KW. Resources: KW, Y-JX, H-MY, Y-QC, and Z-HL. Investigation: J-YZ, SF, H-FZ, W-WP, Q-ZN, J-JL, CL, H-KZ, and YM. Formal analysis: KW, Y-JX, H-MY, Y-QC, Z-HL, and J-YZ. Writing–original draft: All authors. Writing–review & editing: WL and S-QC. All authors contributed to the article and approved the submitted version.

## Supplementary material

Supplementary Figure 1 Kaplan-Meier curves for **(A)** overall survival, and **(B)** progression-free survival for the high-risk patients. High-risk status was defined as hepatocellular carcinoma with portal vein tumor thrombosis extending to the main portal vein and/or the superior mesenteric vein, and/or tumor ≥50% of the liver.

Supplementary Figure 2
**(A)** Treatment response of efficacy-evaluable patients stratified by tumor mentational burden (high v low). (B) Distribution of genetic variations associated with treatment response. In one of the responding patients, none of the above genes were altered.

Supplementary Figure 3 Kaplan-Meier curves for **(A)** overall survival, **(B)** progression-free survival, and **(C)** time to progression for the tumor mutation burden subgroups.
