# Supplementary figures and images for: Intensity-modulated radiotherapy combined with systemic atezolizumab and bevacizumab in treatment of hepatocellular carcinoma with extrahepatic portal vein tumor thrombus: A preliminary multicenter single-arm prospective study
Source: Front Immunol. 2023 Feb 16;14:1107542. doi: 10.3389/fimmu.2023.1107542 (PMC9978499; doi:10.3389/fimmu.2023.1107542)

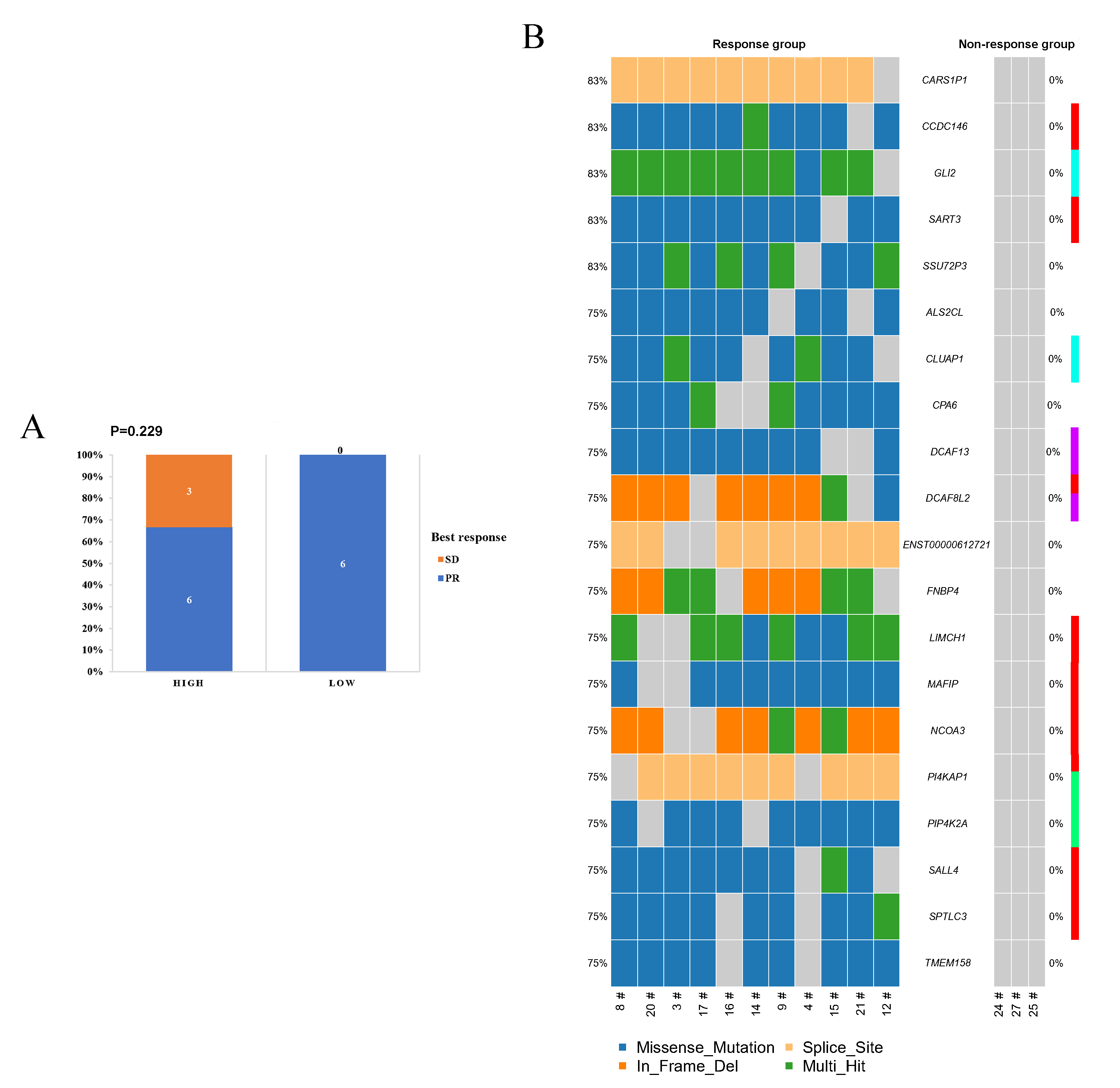

Supplement: Supplementary Figure 1 — Kaplan-Meier curves for (A) overall survival, and (B) progression-free survival for the high-risk patients. High-risk status was defined as hepatocellular carcinoma with portal vein tumor thrombosis extending to the main portal vein and/or the superior mesenteric vein, and/or tumor ≥50% of the liver. [file Image_1.tif]

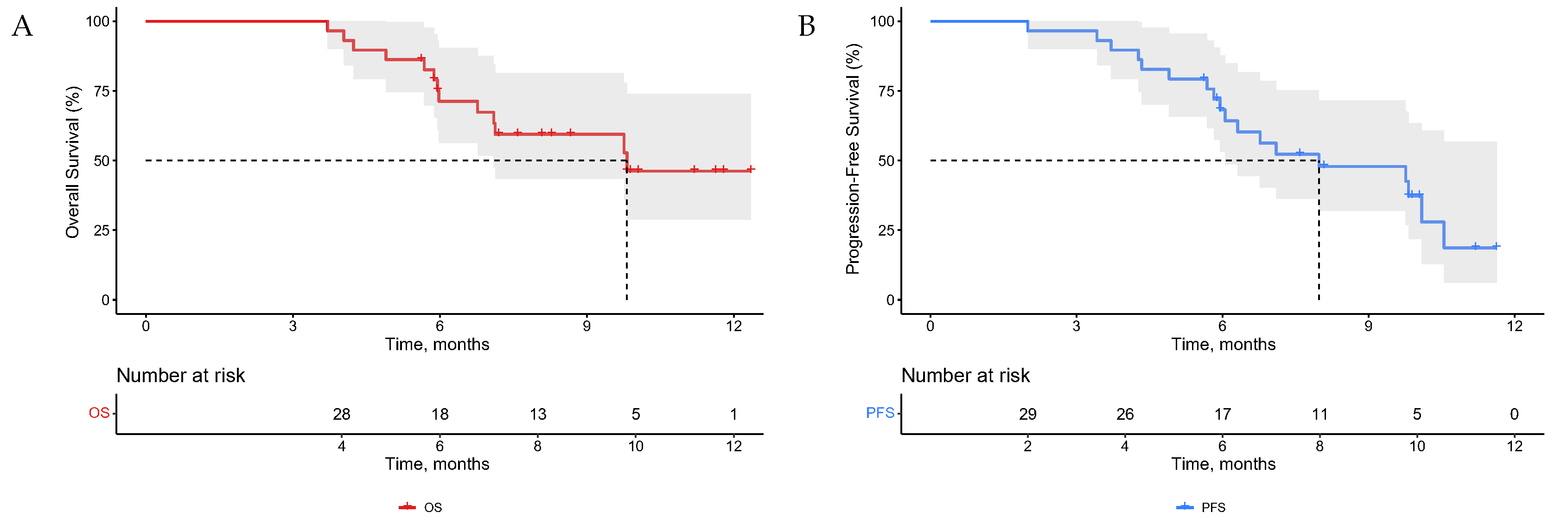

Supplement: Supplementary Figure 2 — (A) Treatment response of efficacy-evaluable patients stratified by tumor mentational burden (high v low). (B) Distribution of genetic variations associated with treatment response. In one of the responding patients, none of the above genes were altered. [file Image_2.tif]

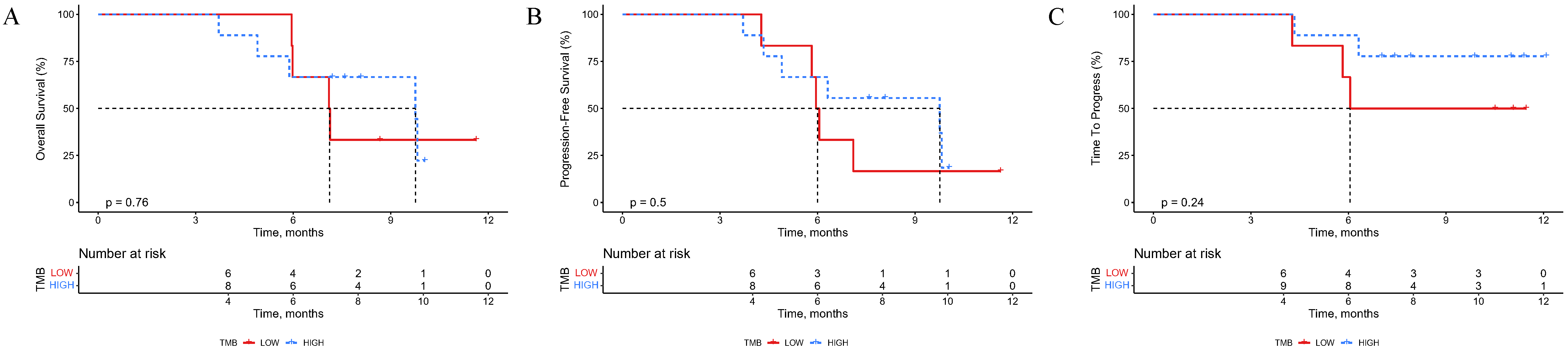

Supplement: Supplementary Figure 3 — Kaplan-Meier curves for (A) overall survival, (B) progression-free survival, and (C) time to progression for the tumor mutation burden subgroups. [file Image_3.tif]
