# Supplementary material for: Intensity-modulated radiotherapy combined with systemic atezolizumab and bevacizumab in treatment of hepatocellular carcinoma with extrahepatic portal vein tumor thrombus: A preliminary multicenter single-arm prospective study
Source: Front Immunol. 2023 Feb 16;14:1107542. doi: 10.3389/fimmu.2023.1107542 (PMC9978499; doi:10.3389/fimmu.2023.1107542)
Supplement: Supplementary file 4 [file Table_1.docx]

Supplemental Table 1. Suspected cause of death

| Patients | 3 | 5 | 8 | 9 | 12 | 14 | 19 | 20 | 21 | 23 | 25 | 28 | 30 |
| --- | --- | --- | --- | --- | --- | --- | --- | --- | --- | --- | --- | --- | --- |
| Progression of disease | - | +++ | - | - | - | - | - | + | - | - | +++ | +++ | +++ |
| RT toxicity | - | - | - | + | - | - | - | - | - | - | - | - | - |
| Atezo/bev toxicity | + | - | + | + | - | - | - | - | - | + | - | - | - |
| Tumor-related liver failure | ++ | - | ++ | +++ | ++ | ++ | ++ | ++ | +++ | ++ | - | - | - |
| RT-related liver failure | - | - | - | - | - | - | - | - | - | - | - | - | - |

RT, radiotherapy; Atezo/bev, atezolizumab plus bevacizumab; -, not related; +, unlikely related; ++, possibly related; +++, definitely related.
